# Spatial Heterogeneity Analysis: Introducing a New Form of Spatial Entropy

**DOI:** 10.3390/e20060398

**Published:** 2018-05-23

**Authors:** Chaojun Wang, Hongrui Zhao

**Affiliations:** 3S Center, Tsinghua University; Institute of Geomatics, Department of Civil Engineering, Tsinghua University, Beijing 100084, China

**Keywords:** information entropy, landscape configuration, thermodynamics, spatial diversity, proximity

## Abstract

Distinguishing and characterizing different landscape patterns have long been the primary concerns of quantitative landscape ecology. Information theory and entropy-related metrics have provided the deepest insights in complex system analysis, and have high relevance in landscape ecology. However, ideal methods to compare different landscape patterns from an entropy view are still lacking. The overall aim of this research is to propose a new form of spatial entropy *(*H_s_) in order to distinguish and characterize different landscape patterns. H_s_ is an entropy-related index based on information theory, and integrates proximity as a key spatial component into the measurement of spatial diversity. Proximity contains two aspects, i.e., total edge length and distance, and by including both aspects gives richer information about spatial pattern than metrics that only consider one aspect. Thus, H_s_ provides a novel way to study the spatial structures of landscape patterns where both the edge length and distance relationships are relevant. We compare the performances of H_s_ and other similar approaches through both simulated and real-life landscape patterns. Results show that H_s_ is more flexible and objective in distinguishing and characterizing different landscape patterns. We believe that this metric will facilitate the exploration of relationships between landscape patterns and ecological processes.

## 1. Introduction

Distinguishing and characterizing different landscape patterns are among the primary concerns of quantitative landscape ecology, since the distributions of energy, material, and species in landscapes are determined by specific patterns [1,2,3,4,5]. Landscape patterns are characterized by both their composition and their configuration, which collectively define landscape structure [6,7]. The interplay between landscape patterns and ecological processes is profoundly important, with patterns constraining processes and processes creating patterns in a reciprocal feedback [8,9,10]. Therefore, accurately capturing and characterizing landscape patterns are the key foundations for most analysis in landscape ecology [3,9].

Among all of the fields of natural sciences, the field of information theory and entropy-related metrics have provided the deepest insights into complex system analysis, and have high relevance in landscape ecology [10,11,12,13]. Descriptions of landscape patterns, dynamics of ecological processes, and interactions of pattern-process across scales in space and time are all constrained by entropy and the second law of thermodynamics [10]. The form of the entropy concept that is widely used in landscape ecology was proposed by Shannon in the 1940s [14]. Shannon entropy (also called information entropy) is a quantitative measure of the diversity and information content of a signal, and it has formed the cornerstone of information theory [15]. After its origination, Shannon entropy was rapidly introduced to the field of landscape ecology (e.g., see literature [16,17]). Several researchers have explored how Shannon’s quantitative theory principles can be applied to space (see literature [2,18,19,20,21,22]). The challenge of this extension of Shannon entropy to spatial analysis is due to the fact that space is a specific form of multi-dimensional system where the different dimensions are intimately linked [23], while information systems that were studied by Shannon are made of messages decomposed into one-dimensional signals [24]. These heuristic studies have clarified some quantitative aspects of landscape patterns. However, one intriguing question is whether or not the notion of diversity that is defined in information theory is influenced by some of the fundamental properties that space generates and conveys [23]. More specifically, when Shannon entropy is applied in space, the problem of dimension mismatch occurs [25].

Fortunately, to date, at least two alternative approaches have been developed to solve this problem. The first was proposed by Claramunt [23]. This approach is extended from Shannon entropy, and derived from the principles of the Tobler’s First law (TFL) in geography. Claramunt’s seminal work is thought-provoking, and it differs from conventional methods by modeling distance as a key factor that influences the way that similar or different entities are interrelated in space [23]. Thus, this entropy-related measure can effectively distinguish different landscape patterns to some degree. However, our experience using this approach shows that it is quite complicated since several kinds of distance need to be calculated. Moreover, as many scholars have argued, distance is not always the key factor that relates to entities in space (please refer to [26,27,28]).

The second approach is based on Boltzmann entropy (also called configuration entropy). Cushman has proposed that the entropy of a landscape mosaic can be calculated using the Boltzmann equation, with entropy equals to the natural logarithm of the number of unique configurations of a landscape (microstates), which has the same total edge length (macrostate) as the focal landscape [3]. This approach can also distinguish different landscape patterns with various macrostates, and it provides a means to understand the relationships between entropy and landscape configurations. However, as the dimensionality and number of categories increase (e.g., realistic landscapes), the number of unique configurations rapidly becomes intractably large, which makes it impossible to calculate the configuration entropy. At the same time, as the dimensionality and the number of categories increase, the number of potential macrostates (i.e., total edge length) also increases extremely rapidly, which makes the calculation more complicated. More importantly, the assumption behind this approach, that true thermodynamic relationships between landscape configuration and entropy, is still questionable.

Thus, methods that effectively describe and distinguish different landscape patterns from an entropy perspective are still lacking. The overall aim of this paper is to propose a new form of spatial entropy (H_s_), which can be used to distinguish and characterize different landscape patterns. H_s_ is an entropy-related index based on information theory, and it integrates proximity as the key factor that relates entities in geo-space. Proximity has been recognized as the central organizing principle in space [27,28], and contains two aspects, including edge length and distance. In this way, H_s_ provides a novel way to study spatial structures of landscape patterns where categories and proximity are both relevant to the analysis. We tested the performance of H_s_ and other similar approaches based on both simulated and real-life landscapes, and our results show that H_s_ is more flexible and sensitive in characterizing and distinguishing different landscape patterns.

## 2. Methods

**Figure 1:**
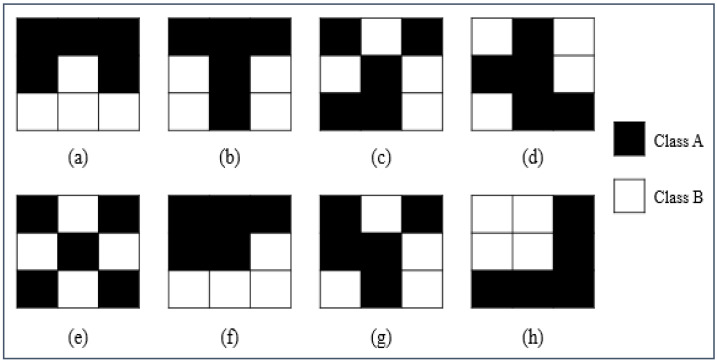
Eight possible configurations (**a**–**h**) of a landscape mosaic with dimensions 3 × 3, two classes, 5 cells of class A, and 4 cells of class B, the length and width of each cell are unit size. Landscapes (**a**–**h**) show different patterns, however, have the same Shannon entropy since all of them contain the same compositional information (based on descriptions and discussions in [3]).

The central focus on spatial heterogeneity has been widely recognized as the salient characteristics of landscape ecology [4], and distinguishing different landscape patterns is the first step in the analysis of spatial heterogeneity. Obviously, Shannon entropy, as discussed above, does not provide sufficient information in spatial analysis since it only captures the compositional information (i.e., richness and evenness) of landscapes, and it ignores the configurational information (see Figure 1 as an example). What would be more useful is to measure the entropy of different landscape patterns at a particular number of classes, and proportionality of each class [3]. Thus, a fundamental question needs to be addressed, is how to evaluate the role that is played by space?

In order to quantitatively measure the influence of space, the fundamental properties that space generates and conveys should be considered. Similarly, to the definition in the context of information theory, a spatial measure of diversity should take influence of space into account when considering the degree of uncertainty in selecting some entities of interest [23].

The First Law of Geography (also called Tobler’s First Law, TFL) may help to solve this problem and to bridge the gaps between Shannon entropy and a way that diversity would be evaluated in space. This law states that “Everything is related to everything else, but near things are more related than distant things” [29], and it has resonated strongly in geography since near and related are useful concepts at the core of spatial analysis and modeling [26,28]. However, the concepts of near and related were vaguely defined by Tobler, which has led to a lot of controversies about what is near and what is related in practical applications (see [27]). Recently, more and more scholars, including Tobler himself, tend to use the concept of “proximity” to measure the nearness between entities in space [30]. Proximity contains two factors, including the total edge length (it measures the total amount of edges between different classes in a landscape, see [3]) and distance, and it is a more flexible and powerful concept in spatial analysis and modeling. Specifically, proximity is proportional to the total edge length between the different classes and is inversely proportional to the distance between different class centers in space [28]. Taking Figure 1 as an example, these eight different configurations have the same Shannon entropy, however, their proximity information are various (e.g., the total edge length of landscapes a–c are 5, 6, 9; and, the distance between different class centers are 1.35, 0.9, 0.45, respectively). Intuitively, proximity contains richer information (both edge length and distance), and it seems to be an efficient tool to measure the role that is played by space.

Thus, through the introduction of proximity, a new form of spatial entropy (H_s_) can be developed that will better quantify spatial heterogeneity (or diversity, as heterogeneity may be regarded as an essential cause and a consequence of diversity in the context of landscape ecology, please see [9]). This idea rests on two general and objective assumptions: on the one hand, the entropy of a landscape should not only reflect the compositional characteristics of entities, but also the spatial structures of their distributions; on the other hand, such an extension of Shannon entropy should reflect our intuitions when perceiving the configurational properties (i.e., disorder) of a landscape. It also should be noted that, as TFL implies, in closed spatial systems, interactions between different entities occur within the represented space. However, there are no such limitations for an open spatial system, and the evaluation of spatial diversity would be far more complex when compared to closed systems [31]. This study focuses on closed spatial systems, and it explores a way that spatial diversity would be measured given a particular number of classes, and proportionality of each class. Based on these assumptions, a new form of spatial entropy can be defined, as follows:

(1) $$H_{s}=-\overset{n}{\underset{i=1}{\sum }}\frac{L_{i}}{d_{i}}p_{i}\log_{2}p_{i}$$

(2) $$L_{i}=\overset{n}{\underset{\begin{array}{c} k=1\\ i\neq k \end{array}}{\sum }}L_{ik}$$

(3) $$d_{i}=\overset{n}{\underset{\begin{array}{c} k=1\\ i\neq k \end{array}}{\sum }}d_{ik}$$

where *n* is the number of different classes and *p_i_* denotes the proportionality of class *i* in a landscape. $L_{i}$ is the total amount of edges between class *i* and other different classes (or patch types), and it equals to the sum of lengths of all edge segments involving the corresponding class [32]. *d_i_*denotes the sum of average distances between the different class centers. It should be noted that the distance that is considered in H_s_ might be any form that fulfils the metric properties of distance (e.g., Manhattan distance, Euclidean distance, contextual distance, cognitive distance). In this study, we consider the Euclidean distance in its application to geographical space. When different class centroids coincide, *d_i_* can be taken as a relatively small constant (such as 0.5-unit length) in order to avoid the “noise” effect of null values in the calculations.

As mentioned above, H_s_ explicitly models proximity as a key factor that relates entities in space, and it builds the bridge between Shannon entropy and a way that diversity (or heterogeneity) would be evaluated in spatial analysis. H_s_ is semi bounded by the interval [0, +∞], and it directly reflects our intuitions that diversity should increase when the total edge length between different classes increases, as diversity should also increase when the distance between different class centers decreases.

## 3. Experimental Validation

In this section, both simulated and real-life landscapes are used for the validation of this new form of spatial entropy through examining: (1) whether it has the ability to distinguish different landscape patterns; (2) and, whether it can characterize the degree of spatial heterogeneity of these patterns. At the same time, we compare the performance of H_s_ with similar entropy-related metrics. However, as [3] pointed out, there is an array of landscape indices proposed to measure the configurational characteristics of landscape patterns. Fortunately, several literatures (for instance, [33,34]) have presented a critical view of the effectiveness of these configurational metrics. According to the results of these recent studies, we choose two other similar entropy-related metrics (spatial entropy, referred to as H_sc_, proposed by Claramunt [21]; interspersion and juxtaposition index, referred to as IJI, as proposed by McGarigal and Marks [35]) for a comparative purpose. The detailed description of these two metrics can be found in Appendix A. The calculation of these three metrics is based on MATLAB R2017b [36].

### 3.1. Simulated Landscapes for Validation

**Figure 2:**
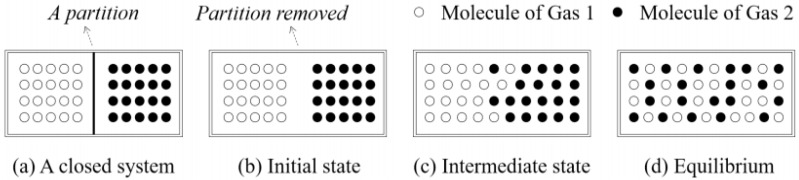
A schematic diagram of ideal gas mixing in a closed system. (**a**) Two kinds of ideal gases are separated by a partition in a container; (**b**) The partition is removed and the ideal gases begin to mix together; (**c**) An intermediate state of the mixture; and (**d**) The final state, equilibrium, of the mixture (after [34]).

Firstly, a set of simulated landscapes is used to evaluate the performances of H_s_, H_sc_, and IJI. The fundamental idea behind the simulation strategy is to create a series of increasingly configurational disordered patterns, and then to examine whether these metrics capture the increasing disorder or not [25]. In order to obtain a sequence of such landscapes, we followed the discussion by [3,34], the mixtures of ideal gases in thermodynamics revisited (see Figure 2). The salient characteristic of gaseous mixtures is that, as [37] noted, during the mixing process, the disorder of the system increases logarithmically until the system reaches its thermodynamics equilibrium, i.e., the maximum degree of disorder.

**Figure 3:**
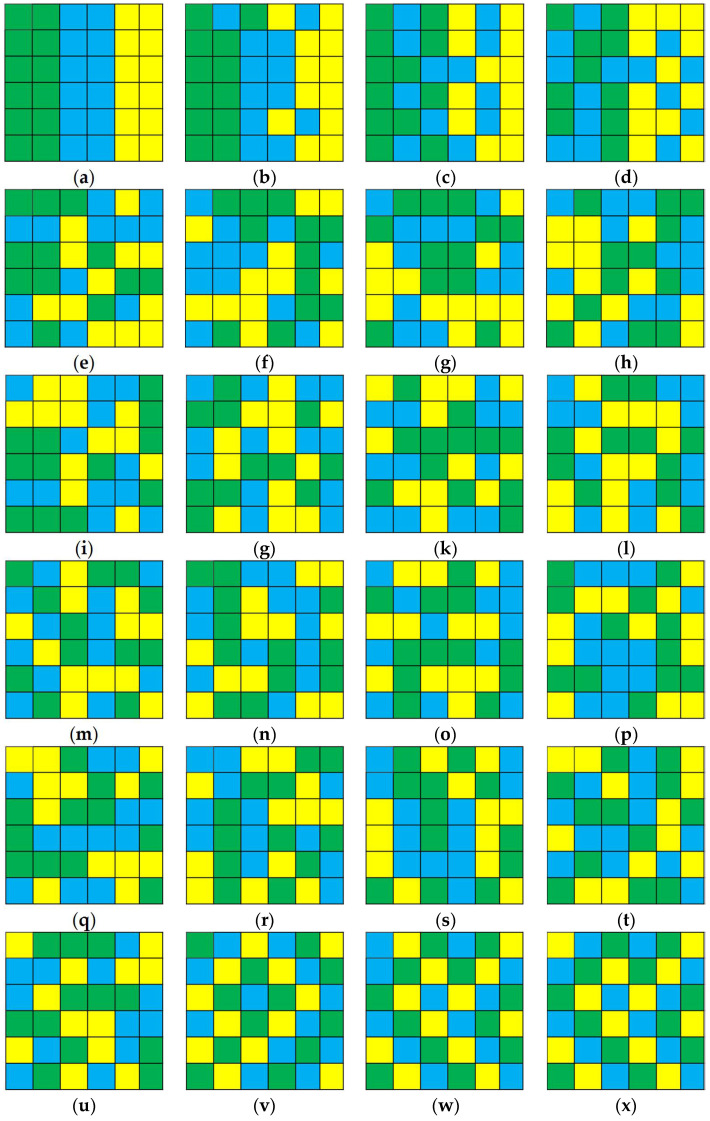
Twenty-four different landscape configurations with dimensions 6 × 6, three classes, and the same proportion of each class. (**a**) An ordered landscape mosaic, like the “Initial state” of gaseous mixtures; (**b**–**w**) Twenty-two increasingly configurational disordered patterns similar to the “Intermediate state” during the mixing; (**x**) The most disorder manner like “Equilibrium state”.

Similar to the process of gaseous mixtures, a set of increasingly configurational disordered patterns can be generated (see [34], and the simulation strategy is presented in Appendix B). Figure 3 shows twenty-four different landscape configurations at a dimensionality of 6 × 6 cells with three classes and the same proportion of each class. However, there will be 36!/(12! × 12! × 12!) = 3.3847e + 15 unique configurations that are generated in this simulation strategy. We choose them for two main reasons: on the one hand, these simple landscape patterns show different configurational information; on the other hand, the increasing degree of disorder (or heterogeneity) would be captured by naked eye. For instance, Figure 3a shows an ordered configuration, like the “Initial state” of gaseous mixtures, and Figure 3d presents a disorder manner of placing the classes in space that is similar to the “Intermediate state” during the mixing, and Figure 3x denotes the most disordered arrangement of entities, like the “Equilibrium state”. In summary, these simple landscapes are useful to address the question that the role played by space should be considered when entropy is used to evaluate the degree of landscape heterogeneity, in the most perspicuous ways [31]. Furthermore, a statistical test (regression analysis) is applied to evaluate the validation of all three metrics, that is to say, whether their values of these different landscape configurations present a valid logarithmic trend [37]. In this study, the coefficient of determination ($R^{2}$) values of the regression model are used to verify the goodness of fit, and if $R^{2}$ is greater than a half, then the regression model can usually be regarded as a good fit [34,38].

**Figure 4:**
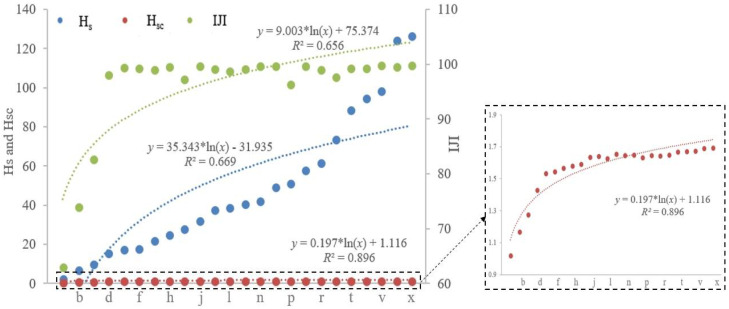
The results of three metrics for twenty-four increasingly configurational disordered landscape mosaics.

The results of three metrics (i.e., H_s_, H_sc_, and IJI) are shown in Figure 4. The regression analysis for each metric is also presented, including regression equation and the coefficient of determination ($R^{2}$).

**Figure 5:**
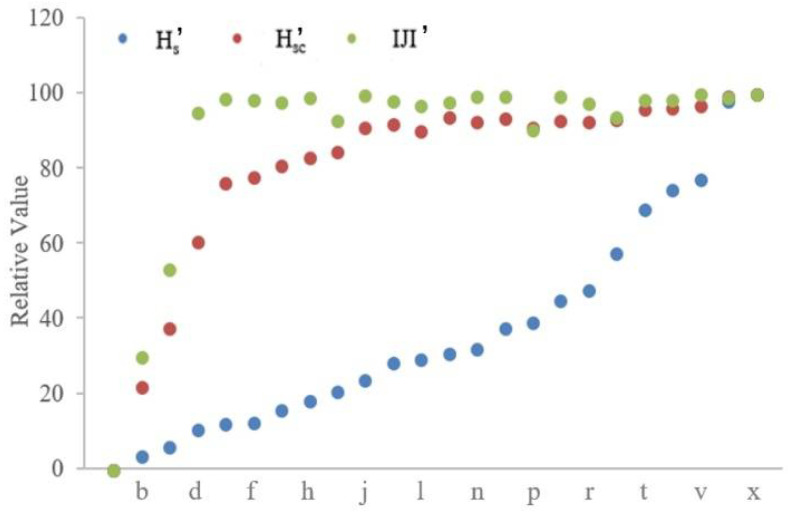
The normalized results of three metrics for twenty-four increasingly configurational disordered landscape mosaics. H_s_’, H_sc_’ and IJI’ denote the normalization of metric H_s_, H_sc_, and IJI, respectively. Taking H_s_’ for an example, H_s_’ = (H_s_ − H_smin_) × 100/(H_smax_ − H_smin_).

The values of three metrics for these different landscape configurations exhibit a valid logarithmic trend over the process of mixing ($R^{2}$ is greater than a half, respectively; Figure 4). Statistically speaking, these three metrics have the ability to distinguish different landscape patterns, and they can characterize the degree of disorder of these patterns. However, the results in Figure 4 also indicate that H_sc_ and IJI are insensitive to the variations among these different patterns; for example, the value of H_sc_ varies from 1.02 (Figure 3a) to 1.69 (Figure 3x) among the twenty-four increasingly configurational disordered patterns; and, the value of IJI changes from 63.09 to 98.18 for Figure 3a–d, however, ranges from 96.51 (Figure 3p) to 100 (Figure 3v,x) for the following twenty patterns. In order to make a direct comparison, we further normalize the results of these three metrics with the same range scope from 0 to 100 (see Figure 5). As Figure 5 shows, the normalized results of IJI are similar among Figure 3d–x (various from 92.95 to 100); and, the relative values of H_sc_ for Figure 3j–x change from 91.13 to 100, which also show little variations. In total, when compared with the performance of H_s_, these two metrics are overall less sensitive to the changes in landscape configurations (Figure 4 and Figure 5).

### 3.2. Real-Life Landscapes for Validation

**Figure 6:**
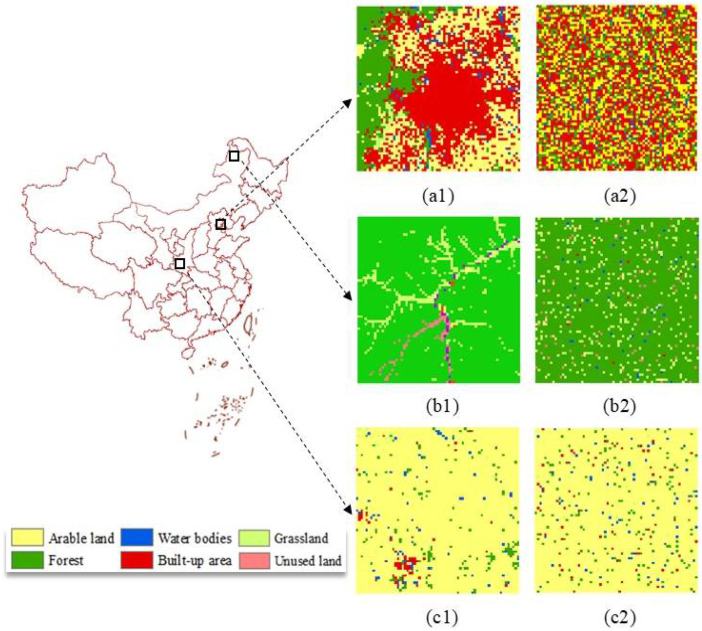
Three pairs of different real-life landscape types. (**a1**) Urban landscape; (**b1**) Forest landscape; and (**c1**) Agriculture landscape. The right one in each pair is randomly reorganized from the left landscape type, respectively. Their main features are shown in Table 1.

Secondly, we also use a set of real-life landscapes for the purpose of validation. This set of data contains three pairs of different landscape types, including urban, forest, and agriculture landscapes, as Figure 6 shows. In each pair, the left one, in fact, is a portion of land-use map of China for 2015, which are provided by Resource Data Center of Chinese Academy of Sciences (http://www.resdc.cn). This land-use map contains six different classes, including forest, water bodies, built-up area, grassland, arable land, and unused land, with a spatial resolution of 1000 m.

The dimension of each landscape type is 70 × 70, and the main features of them are shown in Table 1. The right landscapes in these pairs are randomly reorganized from the left ones. Thus, when compared with the left landscape type in each pair, the right one has the same compositional information, however, at least by visual observation, is more disordered. The results of three metrics regarding to these landscapes are shown in Table 2.

It can be seen from Table 2 that three entropy-related metrics can distinguish these different landscape patterns, and only H_sc_ and H_s_ capture the trend of the configurational disorder of landscapes in each pair (Figure 6; Table 2). However, H_sc_ is less sensitive to the changes in configurations, and its values among these different patterns are similar (see Table 2). IJI fails to depict the degree of configurational disorder of the last two pairs (Figure 6b1,b2,c1,c2, respectively). Only H_s_ can distinguish these different patterns effectively and capture the configurational disorder information sensitively.

## 4. Discussion

Characterizing and distinguishing different landscape patterns have long been the primary concerns of spatial analysis in landscape ecology. In this study, a new form of spatial entropy (H_s_) has been developed to distinguish and to characterize different landscape configurations. H_s_ is an entropy-related index that is based on Shannon entropy, and models proximity as the key factor that relates entities in space. Proximity incorporates both the total edge length and distance, and so it reflects these two important aspects of spatial pattern. In this way, H_s_ provides a way diversity (or heterogeneity) should be evaluated in space. Lower values of H_s_ indicate a landscape pattern with a weaker degree of spatial heterogeneity, like placing entities in an ordered way in space, i.e., entities would be more adjacent to entities of the same class, as TFL describes [29]. In this context, this new form of spatial entropy is similar to the measure of order and disorder that is proposed by Bogaert et al. [39]. When compared with the heuristic work by Batty regarding spatial systems analysis (in [19,21], he developed a derivation of a continuous measure of entropy and applied it to the study the probability distribution over a progressive distance from a given location), H_s_ considers the relative spatial distributions of entities in space.

Both simulated and real-life landscapes are applied to evaluate the performance of H_s_ and similar entropy-related metrics, including H_sc_ (spatial entropy proposed by Claramunt [23]) and IJI (interspersion and juxtaposition index that is proposed by McGarigal and Marks [35]). The results of validation show that all three metrics can distinguish different landscape configurations, and both H_sc_ and IJI are overall less sensitive to changes in landscape patterns, and IJI fails to capture the degree of configurational disorder of these patterns (Figure 3, Figure 4, Figure 5 and Figure 6; Table 2). However, if a landscape metric is insensitive to differences in landscape patterns, it is hard (or fails) to detect landscape structural changes that may be important to understanding ecological processes [40]. The reason why H_sc_ cannot effectively distinguish these different landscape patterns lies in that distance is not always the key factor that relates to entities in space [27,28]. The interspersion and juxtaposition index measures to which extent patch types (or classes) are interspersed, and is a relative metric that denotes the degree of interspersion as a percentage of the maximum possible given the total number of patch types [32], and in fact, it is not a formal entropy metric.

Since accurately describing and characterizing landscape configuration is a key foundation of landscape ecology research [8], H_s_ will help to objectively measure and quantify different landscape patterns. Furthermore, this new form of spatial entropy may be useful to better explain landscape patterns, predict ecological process, and understanding the interactions of pattern-process given that they are all constrained by entropy principles [3,10]. Currently, many scholars concur that landscape ecologists should pay more attention to the linkages between entropy, complexity theory, and landscape ecology as a multiple-scale and hierarchical dissipative structures (see [3,12,41]), and we hope that our new spatial entropy index can contribute to those linkages.

Another application of H_s_, as the experimental results demonstrate, is to measure the degree of order and disorder of a given spatial pattern at the landscape level. This new entropy index captures the configurational disorder of both the simulated and real-life landscapes effectively and sensitively. However, as many scholars’ shrewd observation (e.g., [3,24]), the true thermodynamic relationship between entropy and landscape configuration still needs to be further investigated, for instance, the divergent theoretical assumptions between information and thermodynamic entropies, and the analogy between ideal gases and landscapes. Further research is needed to clarify the true relationships between spatial patterns and thermodynamic disorder, and at the very least, the H_s_ metric that is proposed in this study is highly efficient when compared with similar measures of landscape configurations.

It is necessary to note that this research uses Euclidean distance to calculate H_s_. However, different measures of distance (e.g., Manhattan distance, contextual distance, and cognitive distance) can be considered in the calculation of H_s_, which depends on the specific phenomena studied in landscape ecology [42]. In addition, the simulated landscapes that are presented above are simple landscape patterns produced for validation and comparison among the methods in the most perspicuous ways. The examples (both simulated and real-life ones) that are discussed in this study are regular raster-based landscapes at a particular level dimensionality, number of classes, and proportionality of each class. When H_s_ is applied to more realistic landscapes, higher numbers of classes and proximities make the interpretation of results more complicated since the definitions of the term entropy and its meanings are dialectically vague from different perspectives. Also, when H_s_ is used to irregular and/or vector-based landscapes, the calculation of proximity would be more complicated, and the complex irregular patterns (e.g., polygon with holes) would affect the interpretation of results. Thus, this new form of spatial entropy needs to be further validated on other cases in order to be considered as an efficient and promising method.

## 5. Conclusions

Accurately describing and characterizing landscape patterns are among the core tasks in landscape ecology research. In this study, we introduce a new form of spatial entropy (H_s_), which is extended from Shannon entropy, and is derived from the principles of TFL. H_s_ is an entropy-related index, and it integrates proximity as the key factor that relates entities in space. It provides a novel way to study the diversity of spatial structures of landscapes where categories and proximity are relevant to the analysis. We also tested of the performance of H_s_ and other similar approaches that are based on both simulated and real-life landscapes, and found that H_s_ is more flexible and is sensitive in characterizing and distinguishing different landscape patterns.

We believe future research should focus on the following areas: (1) how this new entropy index would change with dimensionality, number of classes, proportion of each class in the landscape, and the effects of changing scales on the analysis of landscape patterns; (2) how the spatial entropy of landscapes that are represented as irregular vector-based patterns can be calculated; (3) the underlying pattern-process relationships of H_s_ needs to be further explored, which is a quite important issue in landscape pattern analysis [43]; and (4) the connections of H_s_ and other entropy based metrics to true thermodynamic disorder must be further explored and developed.

## Figures and Tables

**Table 1:** Main features of three different landscape types.

Landscapes	Latitude Extent	Longitude Extent	Description
Urban landscape (a1)	38°50′54″–39°28′04″	115°47′08″–116°36′54″	47.1% built-up area, 29.7% arable land, 19.0% forest land, 1.8% grassland, and 2.4% water bodies
Forest landscape (b1)	51°06′38″–51°43′48″	121°59′48″–122°49′34″	89.2% forest land, 0.5% arable land, 7.6% grassland, 0.7% water bodies, 0.2% built-up area, and 1.8% unused land
Agriculture landscape (c1)	38°50′54″–39°37′04″	105°44′53″–106°34′39″	93.2% arable land, 3.3% forest land, 1.6% built-up area, 0.5% grassland, 1.4% water bodies

**Table 2:** The results of three entropy-related metrics of real-life landscapes.

Metrics	a1	a2	b1	b2	c1	c2
H_sc_	1.43	1.75	0.53	0.65	0.42	0.47
IJI	62.06	66.18	42.21	39.79	51.91	47.83
H_s_	43.43	629.34	6.98	54.93	3.05	34.07

Note: H_sc_, spatial entropy proposed by Claramunt [21]; IJI, interspersion and juxtaposition index proposed by McGarigal and Marks [32]; H_s_, the new entropy index proposed in this study. The base of logarithmic in computing each metric is set as 2 in this research, although other bases such as 10 and *e* are available. The detailed descriptions of these real-life landscapes are shown in Figure 6 and Table 1.

## Appendix A

(1) Spatial entropy (*H_sc_*, proposed by Claramunt [21])

Claramunt (2005) introduced the factor of distance and proposed a spatial form entropy based on information theory. The idea behind his research is to consider the primal role of distance that relates entities in geo-space. More specifically, he argues that the entropy should augment when distance between different entities decreases, while the entropy should also augment when the distance between similar entities increases [27]. Thus, this kind of spatial entropy is defined as follows:

(A1) $$H_{sc}=-\overset{n}{\underset{i=1}{\sum }}\frac{d_{i}^{int}}{d_{i}^{ext}}p_{i}\log_{2}p_{i}$$

where $d_{i}^{int}$ means the average distance between the entities of a given class *i* (also called intra-distance); $d_{i}^{ext}$ denotes the average distance between the entities of a given class *i* and the entities of the other classes (also called extra-distance).

(A2) $$d_{i}^{int}=\frac{1}{N_{i}*\left(N_{i}-1\right)}\overset{N_{i}}{\underset{\begin{array}{c} \begin{array}{c} j=1 \end{array}\\ j\epsilon C_{i} \end{array}}{\sum }}\overset{N_{i}}{\underset{\begin{array}{c} k=1\\ k\neq j\\ k\epsilon C_{i} \end{array}}{\sum }}d_{j,k}\;\;if\;N_{i}>1,\;otherwise\;d_{i}^{int}=\lambda$$

(A3) $$d_{i}^{ext}=\frac{1}{N_{i}*\left(N-N_{i}\right)}\overset{N_{i}}{\underset{\begin{array}{c} j=1\\ j\epsilon C_{i} \end{array}}{\sum }}\overset{N-N_{i}}{\underset{\begin{array}{c} k=1\\ k\notin C_{i} \end{array}}{\sum }}d_{j,k}\;\;if\;N_{i}\neq N,\;otherwise\;d_{i}^{ext}=\lambda$$

where$\;C_{i}$ denotes the set of entities of a given class *i*, $N_{i}$ denotes the number of entities of a given class *i*, *N* the total number of entities, $d_{i,j}$ is the distance between two entities *i* and *j*, *λ* is a constant taken relatively small (such as 0.2 or 0.5-unit length) in order to avoid the “noise” effect of null values in the calculations of the average distances [21].

H_sc_ is semi bounded by the interval [0, +∞], and augments when the intra-distance increases, or the extra-distance decreases [21]. For some given intra- and extra-distance values, H_sc_ is maximum when the classes are evenly distributed.

(2) Interspersion and juxtaposition index (IJI, McGarigal and Marks [32])

McGarigal and Marks (1995) introduced the interspersion and juxtaposition index to measure the extent to which the patch type (or classes) are interspersed (i.e., equally adjacent to each other), and lower values characterize landscapes in which the patch types are poorly interspersed. This index (IJI) is computed as follows:

(A4) $$IJI=-\frac{-\sum_{i=1}^{n}\sum_{k=i+1}^{n}\left[\left(\frac{L_{ik}}{L}\right)*\log_{2}\left(\frac{L_{ik}}{L}\right)\right]}{log_{2}\left(0.5\left(n\left(n-1\right)\right)\right)}\left(100\right)$$

where $L_{ik}$ denotes the total length of edge in landscape between patch type (or classes) *i* and *k*, *L* refers to the total length of edge in landscape excluding background, *n* refers to number of classes in the landscape.

IJI increases in value as patch types tend to be more evenly interspersed, and it ranges from 0 to 100 [32]. When all patch types are equally adjacent to all other patch types, IJI reaches its maximum value (100).

## Appendix B

The simulation strategy of those twenty-four different landscape patterns (Figure 3) is based on descriptions and discussions in [34], but much simple (only 6 × 6 cells with three classes and the same proportion of each class are considered). The simulation strategy contains four main steps:

1. set Figure 3a as the “seed” pattern, which is regarded as the initial state of a closed system (an ordered landscape mosaic).
2. then select three cells from middle to the sides in the “seed” pattern, and exchange the position of each cell with randomly selected neighboring cell.
3. repeat Step 2 until the pattern is similar to Figure 3x (the most disordered manner like “Equilibrium state” of gaseous mixtures).
4. then choose twenty-two patterns from the output of Step 2. In this way, we can obtain a set of increasingly configurational disordered patterns. It should be noted that, the choose of these twenty-two patterns may be affected by subjective factors, however, the most important thing is that they should present in an increasingly configurational disordered way, which would be captured by naked eye.

These simulated landscape patterns and related algorithms associated with this research are available on online at https://pan.baidu.com/s/1jgLF2PBinmtQDyczNFehBA.
